# Barriers to Helicopter Emergency Medical Service Operations in Nepal: A Retrospective Study of Mission Cancellations

**DOI:** 10.7759/cureus.109851

**Published:** 2026-05-28

**Authors:** Sanjaya Karki, Callum Bayliss, Sitaram Parajuli, Anish Thakur, Raju R Magar, Yuvraj Basnet

**Affiliations:** 1 Emergency and Pre-hospital Care, Nepal Mediciti Hospital, Kathmandu, NPL; 2 Emergency Medicine, East Lancashire Hospitals NHS Trust, Blackburn, GBR; 3 Emergency and Pre-hospital Care, Hospital for Advanced Medicine and Surgery (HAMS), Kathmandu, NPL; 4 Emergency and Pre-hospital Care, Nepal Mediciti Hospital, Lalitpur, NPL

**Keywords:** air ambulance, air medical transport, helicopter emergency medical services, hems, nepal

## Abstract

Background

Helicopter Emergency Medical Services (HEMS) play an important role in geographically challenging regions such as Nepal, where mountainous terrain, limited road infrastructure, and remote settlements can delay access to advanced medical care. However, mission cancellations remain a significant operational challenge and may reduce system efficiency and timely patient transfer.

Objective

The study aimed to analyse the reasons for HEMS mission cancellations at a tertiary care centre in Nepal and identify the financial, operational, environmental, and patient-related factors influencing HEMS utilisation.

Methods

A retrospective observational study was conducted using HEMS coordination data from Mediciti Hospital, Kathmandu, Nepal, between February 2023 and July 2024. All cancelled HEMS missions during the study period were included. Cancellation reasons were categorised using data from the hospital's call register book and analysed descriptively using frequencies and percentages.

Results

During the 18-month study period, 407 HEMS calls were recorded. Of these, 249 missions (61.1%) were completed, and 158 missions (38.8%) were cancelled. Financial constraints were the most common reason for cancellation (54 missions, 34.18%), followed by miscellaneous operational causes (18 missions, 11.39%), helicopter unavailability (17 missions, 10.76%), and visual flight rules (VFR) limitations (16 missions, 10.13%). Weather-related factors accounted for 12 cancellations (7.59%), while night operation limitations contributed to 13 cancellations (8.23%). Patient-related factors, including preference for another medical centre, non-transferable clinical status, and self-transport, accounted for a smaller proportion of cancellations overall.

Conclusion

Financial and operational limitations were the leading causes of HEMS mission cancellations in this single-centre Nepalese study. Improving financial protection mechanisms, increasing helicopter availability, and expanding night-flight and all-weather operational capability may improve the efficiency and reliability of HEMS delivery in resource-limited mountainous settings. Further multicentre prospective studies are required to better inform sustainable HEMS development in Nepal.

## Introduction

Nepal’s mountainous terrain, limited road infrastructure, and scattered rural settlements present substantial challenges to timely emergency medical care [1]. In this setting, Helicopter Emergency Medical Services (HEMS) play an important role in the rapid transfer of critically ill or injured patients from remote regions to tertiary centres. In Nepal, HEMS capability is currently limited to three tertiary hospitals in Kathmandu: Nepal Mediciti Hospital, Grande International Hospital, and Hospital for Advanced Medicine and Surgery (HAMS)[2,3]. As weather conditions can significantly affect helicopter operations in mountainous regions, contextual climatic conditions are important when interpreting HEMS activity. Weather conditions during the study period were not significantly different from previous years.

Globally, HEMS has demonstrated significant benefits through reduced transfer times and improved outcomes in trauma, cardiac, and obstetric emergencies [4]. However, these operations are resource‑intensive and sensitive to factors such as funding, weather, aircraft availability, regulatory limits, and flight‑safety protocols. These challenges are typically magnified in low‑ and middle‑income countries (LMICs), where aviation infrastructure and healthcare financing mechanisms are underdeveloped [5].

While previous studies have comprehensively examined HEMS utilisation patterns, performance, and cancellation trends in high‑income nations [6,7], there is limited evidence from South Asia and the Himalayan region, where geography and resource constraints uniquely affect operations. A clearer understanding of local HEMS challenges is essential to improve service effectiveness, reduce operational inefficiencies, and guide evidence‑based policy development.

The present study analyses mission cancellation data from Mediciti Hospital, a tertiary care centre in Kathmandu, Nepal. By identifying and quantifying the principal operational, financial, and environmental factors underlying helicopter mission cancellations, the study provides a descriptive overview of systemic barriers influencing HEMS utilisation. The findings may help to inform future discussions on improving the organisation and delivery of air-medical transport services in Nepal.

## Materials and methods

Study design and setting

This retrospective observational study was conducted at Mediciti Hospital, a tertiary care centre in Kathmandu, Nepal, with an established HEMS coordination unit. The hospital coordinates helicopter transfers for both primary scene responses and interfacility referrals. However, the data included focused only on ICU-to-ICU transfer.

Kathmandu is located within a valley at a relatively lower altitude compared with Nepal’s Himalayan and high-mountain regions and experiences less extreme climatic variation. As such, environmental and weather-related operational conditions at the study site may not fully reflect those encountered in other geographically more challenging regions of the country.

Study period and population

The study included all HEMS cancellations recorded between February 2023 and July 2024. During this period, 407 HEMS calls were received. Of these, 249 resulted in completed missions, while 158 (38.8%) were cancelled and included in the analysis.

Operational definitions

HEMS Call: Any request for helicopter emergency medical service received by the coordination unit.

HEMS Mission: A call that proceeded to the activation and mobilisation of a helicopter.

Completed Mission: A mission that resulted in patient transport.

Cancelled Mission: An activated mission that was aborted before patient transfer.

Data collection

Data were extracted from the hospital’s HEMS dispatch register book and operational coordination records maintained by the HEMS coordination unit. These records are completed prospectively by trained HEMS coordinators using a structured standardised dispatch and cancellation recording form.

Cancellation reasons were pre-specified in the register book and included predefined categories such as financial constraints, helicopter unavailability, Night-operation limitations, weather-related restrictions, patient or family preference, clinical non-transferability, and patients transporting themselves.

In addition, a predefined “miscellaneous” category was used to capture cancellations not attributable to a single dominant reason. This category included situations such as sudden changes in clinical or logistical circumstances after activation, communication failures between referring and receiving teams, operational issues (e.g., aircraft technical problems or crew unavailability), adverse environmental conditions not otherwise classified, and instances where post-activation clinical reassessment determined that HEMS transfer was no longer appropriate.

No patient-identifiable information was collected.

Data analysis

Data were analysed using descriptive statistical methods. Frequencies and percentages were calculated to summarise the distribution of cancellation categories. Simple proportion calculations were used to describe the relative contribution of each cancellation reason to the overall dataset. No inferential statistical testing was performed, as the aim of the study was purely descriptive.

Ethical considerations

This study involved a retrospective analysis of anonymised operational data obtained from the HEMS coordination register book at Mediciti Hospital. No patient-identifiable information was collected or analysed. In accordance with institutional policy, formal ethical approval from the Institutional Review Committee was not required due to the retrospective nature of the study and the use of anonymised operational data. The requirement for informed consent was therefore waived.

## Results

During the 18-month study period, a total of 158 HEMS missions were cancelled. Overall, out of 407 HEMS calls, 249 (61.1%) resulted in completed missions, while 158 (38.8%) were cancelled. The distribution of cancellation reasons is presented in Table 1. Overall, system-related and financial factors accounted for the majority of cancellations, while environmental and patient-related clinical factors contributed a smaller proportion.

**Table 1:** Reasons for HEMS Mission Cancellations, Nepal (Mediciti Hospital, Nepal; February 2023 to July 2024) HEMS: Helicopter Emergency Medical Services

Reason	Calls (n)	Percentage (%)
Financial Constraints	54	34.18
Miscellaneous	18	11.39
Helicopter Unavailable	17	10.76
Visual Flight Rules (VFR) Limitations	16	10.13
Patient/Family Preference for an Alternative Centre	14	8.86
Night Operation Limitations	13	8.23
Weather‑related Restrictions	12	7.59
Non‑transferable Clinical Condition	10	6.33
Self-transported	4	2.53
Total	158	100.00

## Discussion

This study provides one of the first institutional analyses of HEMS mission cancellations in Nepal. All missions in this study were interfacility (ICU to ICU) transfers across the Kathmandu Valley, hilly regions, and Terai plains. The term “mountainous environment” is therefore used in a broader contextual sense reflecting national geographic and infrastructural constraints rather than exclusively high-altitude scene operations. No pre-hospital scene missions were recorded.

Overall, cancellations were mainly driven by financial and operational factors, with a smaller contribution from environmental and patient-related causes.

Financial constraints

Financial limitations accounted for more than one-third of all cancellations.

Nepal’s healthcare system relies heavily on out-of-pocket expenditure [8], whereas HEMS in many high-income countries is typically supported through insurance or public funding, reducing financial barriers to activation [9]. Similar patterns have been reported in Austria and the United Kingdom, where patient cost is a rare cause of cancellation due to established reimbursement systems [10,11]. Expanding insurance coverage or introducing targeted subsidies may therefore improve access to HEMS in Nepal.

The cost of helicopter transfer remains substantial, with estimated operating expenses of US$1,300-3,000 per hour [12]. When considered alongside Nepal’s per capita annual income of approximately US$1,456 [13], HEMS is financially inaccessible for many patients. As a result, ground transport often becomes the only feasible option following activation, even when air transfer is initially requested.

Operational limitations

Operational factors were the second most common cause of HEMS cancellation. These included helicopter unavailability, night operation limitations, and flight capability restrictions. Night operation limitations refer to the absence of night-flight or night-vision capability, as well as regulatory and operational constraints in low-visibility conditions.

In Nepal, HEMS operations predominantly rely on light civilian helicopters such as the Airbus AS350 (H125) [14,15], which have limited instrument flight capability and restricted all-weather performance. These inherent technical constraints, combined with limited fleet availability, influence operational thresholds and reduce flexibility during marginal conditions.

Such limitations are well recognised in low-resource and geographically complex settings where aviation infrastructure remains constrained [16,17]. In contrast, many European HEMS systems operate twin-engine aircraft such as the Airbus H135/H145 and Leonardo AW109/AW169, often configured for instrument flight rules (IFR) operations with enhanced avionics, night-vision capability, and de-icing systems. These features allow greater operational reach, although they are also associated with stricter adherence to safety thresholds during adverse conditions [18,19].

Comparable challenges have been described in other mountainous countries, although systems, such as in Norway, benefit from more developed instrument flight capability and structured night-operation services [20]. Strengthening night-flight capability and improving helicopter availability may therefore reduce avoidable cancellations.

Environmental and patient-related factors

Weather-related cancellations accounted for less than 8% of cases. This is substantially lower than the proportions reported in many high‑income settings, where weather contributes between 20% and 40% of HEMS cancellations [21]. This likely reflects conservative pre-dispatch decision-making and the limited all-weather capability of the current fleet, which reduces the likelihood of mission activation under marginal meteorological conditions.

Patient-related cancellations, including self-transport, likely reflect structural rather than preference-driven factors. In these cases, patients were transported by alternative means such as private vehicles, taxis, or police following activation, most often due to financial or logistical constraints.

Non-transferable clinical conditions referred to situations in which, following clinical assessment and discussion between referring teams and the HEMS coordination unit, air transfer was deemed not clinically indicated or feasible. This included patients suitable for local management or cases where transport risks outweighed potential benefit.

Cancellations occurred both before helicopter departure and after activation but before patient pickup, highlighting the dynamic and time-sensitive nature of HEMS coordination in a resource-limited setting.

Implications for practice

Improving financial protection through insurance schemes or subsidies may reduce preventable cancellations. Operational improvements such as expanded night-flight capability, increased helicopter availability, and improved coordination between referring centres may enhance system efficiency.

Refinement of triage processes may also help reduce unnecessary activation and improve resource use.

Limitations

This study was conducted at a single tertiary centre and may not represent national HEMS activity. The retrospective design relied on registry completeness, and some contributing factors may not have been captured. Patient outcome data were unavailable, limiting assessment of clinical consequences. Future multicentre prospective studies with outcome measures are required for a more comprehensive evaluation.

## Conclusions

This single-centre study from Mediciti Hospital, a tertiary hospital in Kathmandu, found that most HEMS mission cancellations were related to financial and operational factors, while environmental and weather-related issues accounted for a smaller proportion. The findings reflect the practical constraints affecting HEMS in this setting, particularly those linked to healthcare financing mechanisms and aviation capacity.

Improving financial protection for patients, for example, through the development of a national insurance scheme, alongside strengthening night-flight capability, all-weather operational readiness, and helicopter availability, may enhance the efficiency and reliability of HEMS in Nepal. However, these findings should be interpreted within the context of a single-centre study and may not fully reflect variation across different regions of the country.

Further multicentre, prospective studies with outcome-based analyses are needed to better inform the development of sustainable HEMS systems in geographically diverse and resource-limited settings such as Nepal.
